# Antimicrobial Activity of Cerium Oxide Nanoparticles on Opportunistic Microorganisms: A Systematic Review

**DOI:** 10.1155/2018/1923606

**Published:** 2018-01-23

**Authors:** Isabela Albuquerque Passos Farias, Carlos Christiano Lima dos Santos, Fábio Correia Sampaio

**Affiliations:** Science Center of Health, Federal University of Paraíba, 58.051-900 João Pessoa, PB, Brazil

## Abstract

An evaluation of studies of biologically active nanoparticles provides guidance for the synthesis of nanoparticles with the goal of developing new antibiotics/antifungals to combat microbial resistance. This review article focuses on the physicochemical properties of cerium oxide nanoparticles (CeNPs) with antimicrobial activity.* Method*. This systematic review followed the* Guidelines for Transparent Reporting of Systematic Reviews and Meta-Analyses*.* Results*. Studies have confirmed the antimicrobial activity of CeNPs (synthesized by different routes) using nitrate or chloride salt precursors and having sizes less than 54 nm.* Conclusion*. Due to the lack of standardization in studies with respect to the bacteria and CeNP concentrations assayed, comparisons between studies to determine more effective routes of synthesis are difficult. The mechanism of CeNP action likely occurs through oxidative stress of components in the cell membrane of the microorganism. During this process, a valence change occurs on the CeNP surface in which an electron is gained and Ce^4+^ is converted to Ce^3+^.

## 1. Introduction

The prevalence of health care-associated infections is high, especially those of blood and urinary tract infections that are associated with catheters and surgical site infections [1].

Microorganisms responsible for such infections include, in order of decreasing frequency,* Staphylococcus aureus*,* Enterococcus *spp., and* Escherichia coli*. Resistant microorganisms are present in 20% of infections, especially methicillin-resistant* Staphylococcus aureus* (MRSA) [1].

The yeast* Candida albicans *is the species most commonly involved in fungal infections [2].* Candida albicans* is a dimorphic and commensal fungus that colonizes the skin, gastrointestinal tract, and reproductive system [3]. The number of new cases of fungal infections in immunocompromised patients is increasing throughout the world [4, 5].

The use of nanotechnology to develop nanoscale materials having antimicrobial activities has been proposed for the development of new therapeutic products and effective strategies for prophylaxis and treatment of infections [6–8]. Recently, nanomaterials exhibited a great potential as contrast agents for visualizing the gastrointestinal tract [9] and nanofibers have also been used as carriers for nanoparticles that can interfere in multidrug resistant bacteria infections [10].

In comparison with small molecule antimicrobial agents, which have short-term activity and are often environmentally toxic, nanoparticulate agents with antimicrobial effects exhibit prolonged effects and are minimally toxic [8]. In addition,* Escherichia coli *bacteria growth inhibition has been shown to be inversely proportional to the size of the nanoparticles [11].

Among these agents, the nanoparticulate cerium dioxide (CeNP) is a rare earth metal oxide of the cubic fluorite type [12, 13] and is of great interest because of its optical and electronic properties. It has extensive industrial applications in medicine, catalysis, and optical and sensor technologies [14]. Its properties are related to its valence, since cerium is the only stable tetravalent state lanthanide, whereas other lanthanides are only stable in the trivalent state [15].

Natural sources of nanoparticles include soil erosion, water evaporation sprays, and plants [16]. In industry, cerium metal is present in sunscreen, solid electrolytes, solar cells, fuel cells, luminescence, photocatalysts, and sensors [17]. Synthesis methodologies attempt to obtain small, high-surface-area particles to potentiate the chemical, physical, and antimicrobial properties of the nanoparticles.

The development of novel antimicrobial agents is of great interest due to the increase in the mortality rate associated with infection [18]. The goal of this systematic review is to address the physicochemical properties of cerium oxide nanoparticles having antimicrobial activity. The evaluation of biologically active nanoparticles provides guidance to nanoparticle synthesis with the aim of developing new antibiotics/antifungals to combat infection.

## 2. Material and Methods

**Figure 1:**
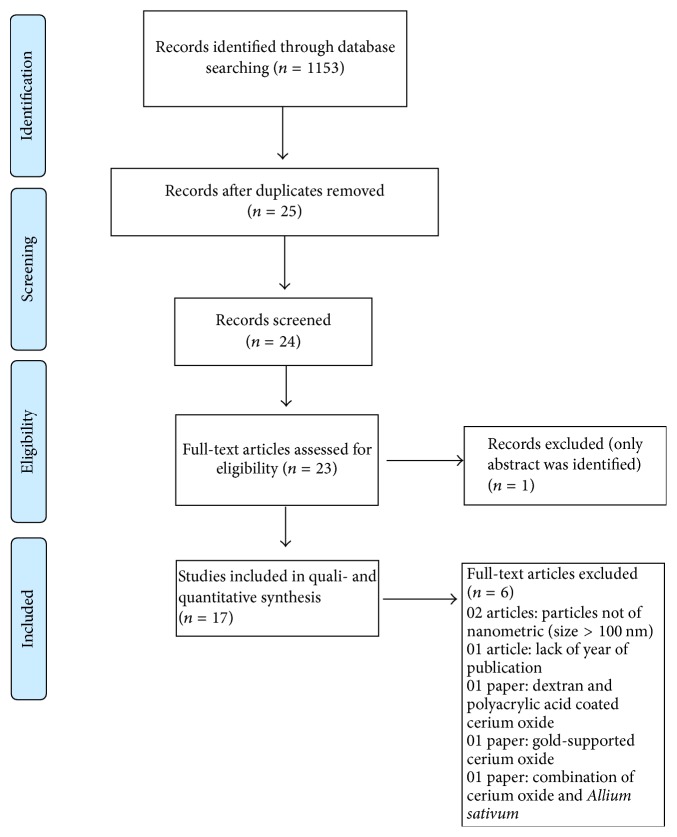
Flow diagram of the search strategy used to identify studies included in this review based on PRISMA guidelines [19].

This systematic review followed the* Guidelines for Transparent Reporting of Systematic Reviews and Meta-Analyses (PRISMA statement)* [19]. The systematic identification of articles was performed in five databases: Google Scholar, SciELO (Scientific Electronic Library Online), PubMed, Lilacs (Latin American and Caribbean Literature on Health Sciences), and Web of Science (Figure 1).

For the retrieval and selection of articles, the following keywords were used: cerium oxide, antimicrobial activity, antifungal activity, bacterial activity, toxicity, and nanoparticles.

All English, Spanish, and Portuguese articles related to the topic that were published from 2006 to October 27, 2016, were selected for analysis. The final selection of articles was made using qualitative criteria in accordance with the theme of cerium oxide nanoparticulate antimicrobial activity. Initially, the titles and abstracts of the articles were assessed by two researchers. Only complete articles were included in the study.

Of the 24 studies identified, seven articles were excluded for the following reasons: the particles were not of nanometric (size > 100 nm) (2 articles), only the abstract was available (1 article), there was a lack of article data (such as year of publication) (1 article), dextran and polyacrylic acid-coated cerium oxide was utilized (1 article), gold-supported cerium oxide was utilized (1 article), and a combination of cerium oxide with* Allium sativum *was utilized (1 article). Thus, a total of 17 articles composed the final sample.

## 3. Results and Discussion

### 3.1. Cerium Oxide Nanoparticle: Synthesis and Physicochemical Characteristics

The materials used during nanoparticle synthesis influence the size and shape of the resulting nanocrystals. According to the* in vitro* studies analyzed in Table 1, there are a variety of CeNP synthesis routes, with the predominant one using ammonium cerium as the precursor salt. Due to solubility, nitrate is preferable to other salts, and it also results in a homogeneous solution [17]. However, cerium chloride salt forms residual chlorine, which does not adversely affect biological systems and therefore is potentially the best precursor material for biological applications [15].

Of the 17 evaluated studies of CeNP antimicrobial activity (Table 1), seven used the principle of green chemistry with extracts of plants, fruits, and fungi [13, 14, 17, 20–23]. The green synthesis route is considered important because it is nontoxic and of low cost and decreases the use of substances that are harmful to human health and the environment [14, 22].

The morphology of the nanoparticles was determined by transmission electron microscopy (TEM) in the majority of studies (Table 1). The shapes of the particles observed through transmission electron microscopy were elliptical, spherical, square, oval, rectangular, triangular, and irregular. Microscopy was also used to evaluate the size of nanoparticles, which ranged from 5.0 [21] to 54 nm [13].

The average size of the CeNP crystallite was estimated by the Debye-Scherrer formula, as 58.82% (*n* = 10) of the analyzed articles used this method. However, there are cases of divergence between these results and those observed using TEM. For example, a 24 nm size value calculated by the formula method was observed to be approximately 5 nm using TEM [21]. Regardless of the method, the analyzed studies highlighted the antimicrobial activity of nanoparticles at a particle size of less than 100 nm [24].

The Debye-Scherrer formula uses X-ray diffraction data, specifically the width at half-maximum of the diffraction peak [15, 17]. The size of the nanoparticle can also be gauged by a formula that uses data from the Brunauer-Emmett-Teller (BET) equation, which considers the specific surface area and density of the nanoparticles [25].

X-ray diffraction was used in studies to confirm the face-centered cubic crystalline structures. The absence of peaks from structures other than the nanoparticulate object of analysis indicates purity of the synthesis product [13].

Surface area is relevant because it is inversely proportional to the nanoparticle size [26, 27]. Smaller crystal sizes and higher surface area lead to higher antibacterial activity. This physical characteristic was described in only three studies [28–30]. The smaller nanoparticles were those with the largest areas favoring a large catalysis surface area [26, 27].

The potential for CeNP catalysis is also influenced by the valence state of Ce^4+^ or Ce^3+^ [31]. This feature directly influences the anti- or prooxidant potential of CeNPs and determines different responses of the substrate to processes such as oxidative stress, superoxide radical cleaning, and hydrogen peroxide production. The conversion of Ce^4+^ to Ce^3+^ was observed in* Escherichia coli* [29], J774A.1 macrophage cells [31], and the hippocampus and cerebellum [32]. The surfaces of algae cells are protected against reactive oxygen species (ROS) in the face of low amounts of Ce^3+^ and high amounts of Ce^4+^. The autoregenerative mechanism of valence reversion influences the protective or toxic effect of the nanoparticle [33].

### 3.2. Studies of Cerium Oxide Nanoparticles against Opportunistic Microorganisms

The inhibitory activity of CeNP on microbial growth was studied in Gram-positive and Gram-negative planktonic bacterial cultures and biofilms. Microbiological tests used to test CeNP activity included enumeration of colony forming units (CFU), agar diffusion, time-kill, and cell viability using fluorescence assays (Tables 2–7).

Agar diffusion was the most frequently used to evaluate the sensitivity of* S. aureus* (Gram-positive) to CeNP, being used in 10 studies involving* S. aureus* (Table 2). The NCIM-5022 strain was tested in three studies [17, 22, 30] and showed little sensitivity to CeNP (diffusion halos between 0.53 and 3.33 mm). In contrast, another study [13] showed the formation of a 17 mm of halo, but information concerning the strain and nanoparticle concentration was omitted. For time-kill tests, a greater than 50% inhibition of the* S. aureus* at concentrations of 2.58–3.44 mg/mL [15] was observed. The two studies with the most significant antimicrobial activity results for* S. aureus* had cerium chloride as the CeNP precursor agent and a particle size of less than 54 nm in common (Tables 1 and 2).

The macrodilution test method detected a CeNP minimal inhibitory concentration (MIC) of 50 ± 10 *μ*g/mL using a planktonic culture of* E. coli* (Gram-negative) and 90 ± 40 *μ*g/mL for a biofilm; CeNP was sonicated prior to treatment for 1 h [34]. The MIC values for biofilms were superior to the planktonic culture, probably since the biofilm is more conducive to microorganism development. The benefits of antimicrobial nanoparticles have been suggested to be above and beyond other molecules because of their ability to penetrate biofilm substrates. Krishnamoorthy et al. [18] reported the lowest MIC of 16 *μ*g/mL against* E. coli *for CeNP synthesized from cerium nitrate using sonochemical method and particles ≤ 25 nm in size (Table 1).

Sonication to avoid the formation of nanoparticle agglomerates is a relevant factor, as is the use of surfactants to form micelles around the nanoparticles. CeNPs made with Tween 80, Triton X114, and polyvinylpyrrolidone surfactants at concentrations of 0.01 and 0.001% (p/v) were tested for inhibition of* E. coli.* The lowest concentration observed with the highest sensitivity was 0.001% with Tween 80, indicating the lowest required surfactant concentration for generating micelles around the nanoparticles [16]. In addition, it is believed that the surfactant changes the surface charge of CeNP, forming a complex with cerium, which is capable of filling the oxygen vacancy and thus prevents the antioxidant effect [16].

The antimicrobial activity of CeNP is concentration dependent [11, 15, 20]. Zeyons et al. [29] also observed this for* E. coli* by enumerating CFUs; however, the result was not dose dependent for the viability test using fluorescence. In the fluorescence assay, the positively charged dye penetrates the altered membrane of the microorganism when interacting with a negatively charged material. Positively charged nanoparticles in large quantities around the cell will interfere with the action of the dye; thus, the CFU count method is more consistent for verifying the ability of the cells to form colonies.

For* Pseudomonas aeruginosa *(Gram-negative), only three studies evaluated its sensitivity to nanoparticles, with MICs of 20 ± 5 *μ*g/mL and 70 ± 0.0 *μ*g/mL for planktonic cultures and biofilms, respectively [34]. The formation of an inhibition zone ranged from approximately 3 mm to 4.67 mm (Table 4). The results of Ravishankar et al. [30] showed a greater CeNP activity against* P. aeruginosa* in smaller doses when the particles were synthesized by combustion and cerium ammonium nitrate was used as a precursor.

*Bacillus subtilis* (Gram-positive) was sensitive to CeNP, with an inhibition greater than 50% observed [23]; however, for the 1 mg/mL concentration, there was no inhibition zone formation (strain not reported) [20], although the CIM observed by Krishnamoorthy et al. [18] was quite small (4 *μ*g/mL) for the KACC strain 14394 (Table 5). This difference can be attributed to differences in strain, route of synthesis, and the salt precursor used.

The genus* Proteus* (Gram-negative) was tested in four of the 17 studies analyzed. The formation of a ~3-mm inhibition zone was observed for* Proteus vulgaris* [20, 21], and the inhibition zone was 11.0 ± 0.51 mm for* Proteus morganii *[35] (Table 6).

*Streptococcus pneumoniae* (Gram-positive) showed a sensitivity to the CeNP at a concentration of 5 mg/mL with the formation of a 3.33 ± 0.33 mm inhibition zone [20] (Table 7).

Only one study evaluated the sensitivity of* C. albicans* to CeNP [7] using a clinical strain (UCM Y-690). A CeNP concentration of 0.017 mg/mL (lowest) yielded a reduction in the viability of the fungus, while a 0.17 mg/mL concentration caused the complete inhibition of the fungus viability.

An 8 *μ*g/mL MIC of CeNP was observed for* Enterococcus faecalis* (Gram-positive) (KACC 13807) and for* Salmonella typhimurium* (KCCM 40253) [18]. In another* E. faecalis* strain (ATCC 19433), the MIC was 50 ± 20 *μ*g/mL and 270 ± 0.0 *μ*g/mL for the planktonic culture and biofilm, respectively [34]. Other strains showed different MICs; however, the salt precursor was the same, and the synthesis method varied between the studies, with the sonochemical method seeming to be the most effective.

*Klebsiella pneumoniae* (Gram-negative) (ATCC 13833) presented sensitivity to CeNP (MICs of 140 ± 0.0 and 360 ± 160 *μ*g/mL for planktonic culture and biofilm, resp.) [34]. Arumugam et al. [21] observed inhibition zones of approximately 2.6 and 4.67 mm at concentrations of 50 and 100 mg/paper disk, respectively. Similar values were found for* Shigella dysenteriae* (Gram-negative).

The clinical strain urinary tract bacteria* Klebsiella* sp. (6.00 ± 0.74 mm) and* Enterobacter sp*. (6.00 ± 0.12 mm) had the same inhibition zone value [35].

The cerium oxide formed a small inhibition zone (<3 mm) at a concentration of 10 mg/mL for* Klebsiella aerogenes* (Gram-negative) (NCIM-2098) [17, 22]. The nanoparticles produced by Malleshappa et al. [22] were smaller and had a defined shape (cube), while those of Reddy Yadav et al. [17] were larger (when compared with nanoparticles of [22]) with irregular shapes (Table 1).

*Shewanella oneidensis* MR-1 (Gram-negative) is a facultative bacterium and was not sensitive to CeNP at concentrations of 50 to 150 mg/mL [11].

Masadeh et al. [34] performed a study aiming to determine the MIC of CeNP against various Gram-positive and Gram-negative bacteria. Below are the sensitivity of the microorganisms tested for the first time in the literature with CeNP in planktonic culture and biofilms, respectively:* Acinetobacter baumannii* (Gram-negative) ATCC 17978 (70 ± 0.0 *μ*g/mL and 360 ± 160 *μ*g/mL);* Streptococcus pyogenes* (Gram-positive) ATCC 19615 (30 ± 10 *μ*g/mL and 70 ± 0.0 *μ*g/mL);* Haemophilus influenzae *(Gram-negative) ATCC 29247 (360 ± 160 *μ*g/mL and 530 ± 0.0 *μ*g/mL);* Staphylococcus epidermidis* (Gram-positive) ATCC 12228 (20 ± 5 *μ*g/mL and 90 ± 40 *μ*g/mL);* Enterobacter aerogenes* ATCC 29751 (70 ± 0.0 *μ*g/mL and 140 ± 0.0 *μ*g/mL);* Citrobacter freundii* (Gram-negative) ATCC 8090; and* Enterobacter cloacae* (Gram-negative) ATCC 13047 (70 ± 0.0 *μ*g/mL and 220 ± 80 *μ*g/mL for both bacteria).

Kannan and Sundrarajan [13] suggested that CeNP can be used as an effective inhibitor in antimicrobial control systems. The effectiveness of the nanoparticles depends on their morphology and size. Masadeh et al. [34], after testing various strains of different species of microorganisms, stated that CeNP is not a good antibacterial candidate.

### 3.3. Cerium Oxide Nanoparticles against Opportunistic Microorganisms: Mechanism of Action

CeNP showed activity in Gram-positive and Gram-negative bacteria, with the greatest antimicrobial activity observed against Gram-negative bacteria* (E. coli)* [22]. Gram-positive bacteria have a thick layer of peptidoglycan that contains linear polysaccharides chains with short peptides that together form a rigid structure that is difficult to penetrate with CeNP. Gram-negative bacteria have a thin layer of peptidoglycan and a lipopolysaccharide that protects the cytoplasmic membrane from outside chemical agents [22]. Gopinath et al. [20] stressed that the greater antibacterial activity of CeNP on Gram-positive bacteria is possibly because the peptidoglycan layer possesses teichoic acid as interaction site for CeNP. Both studies used the agar diffusion method, which yielded small inhibition halo values at the concentrations tested.

Transmission electron microscopy showed that the cerium oxide nanoparticles with antimicrobial activity against* E. coli *adsorb to the bacteria surface but do not penetrate the cell [11]. These findings are in accordance with Thill et al. [28], who suggested three types of interaction between bacteria and CeNP: (1) adsorption, (2) oxi-reduction, and (3) toxicity.

1. Adsorption occurs by electrostatic attraction, possibility modifying cellular transport via ionic pumps [28]. Extracellular polymeric substances production by a microorganism, for example,* Synechocystis*, can compromise adsorption and the consequent oxi-reduction [29].
2. In the process of oxi-reduction, modifications occur on the surface of the nanoparticle and the bacteria. The Ce^4+^ charge of the nanoparticles is reduced to Ce^3+^ in the presence of the bacteria* (E. coli)*, resulting in oxidative stress on lipids and/or proteins in the plasma membrane of the microorganism, or through cellular metabolism electron uptake. It is important to highlight that no reduction of Ce^4+^ was observed in abiotic culture medium [28, 29].
3. Toxicity involves the impairment of cellular respiration, as observed by differences in gene expression, in nanoparticulate exposed and nonexposed* E. coli*. The low level of succinate dehydrogenase and cytochrome b terminal oxidase gene expression in the experimental group indicates that cerium attacks electron flow and bacterial respiration [11]. With respect to* Candida albicans*, it is believed that the interaction between cerium and components of the fungal cell wall can cause irreversible changes, such as blocking fungal enzymatic activity [7].

Another relevant factor in antimicrobial activity is altering of nanoparticle surface charge by the culture medium pH. The extreme pH ranges after the incubation period contribute to this activity by establishing an unfavorable environment for the proliferation by microorganisms [11].

**Figure 2:**
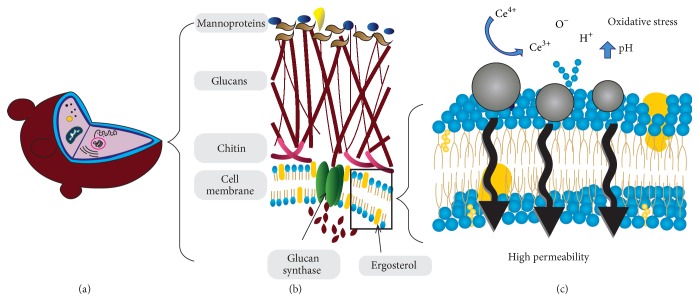
Diagram of the probable mechanism of antimicrobial action for cerium oxide nanoparticulates on the cell membrane.* Candida albicans*; (b) the cell wall of the fungus formed by monoproteins, insoluble glycan and chitin. Phospholipid bilayer of the cell membrane with glycan synthase and ergosterol. (c) Adsorption of cerium oxide nanoparticles, reduction of Ce^4+^ to Ce^3+^, elevation of pH, and oxidative stress of the fungus.

Considering the above factors, a diagram representing the probable mechanism of antimicrobial action for cerium oxide nanoparticles is proposed (Figure 2).

## 4. Conclusions

The reviewed studies report the antimicrobial activity for CeNP as synthesized by different routes that use nitrate or chloride salt precursors and have a size of less than 54 nm. A lack of standardization between the studies, for both the bacteria used and concentrations of CeNP tested, makes them difficult to compare and determine the most efficient synthesis route. Aggregation of CeNP particles by moisture in the air seems to inhibit antimicrobial activity, and it is necessary to standardize the studies with a storage protocol in a dryer, sonicate the nanoparticles, and use Tween-80 surfactant.

The antimicrobial mechanism of action is probably due to oxidative stress on components of the microorganism cell membrane, manly of Gram-negative and fungi microorganisms. This process occurs during CeNP adsorption to the bacterium, which is favored by the acidic pH of the site of infection, since at a low pH, the nanoparticles become positively charged and more easily adhere to the negatively charged bacteria through electrostatic interactions. During this process, a change in valence on the surface of the cerium oxide nanoparticle occurs by gain of an electron, converting Ce^4+^ to Ce^3+^. The greatest antimicrobial activity observed against Gram-negative and fungi occur probably by direct contact and unbalance of the outer membrane. Conversely, in Gram-positive bacteria a thick layer of peptidoglycan in their membrane can modulate this effect. As a result, few particles of Ce^4+^ are reduced to Ce^3+^ and the oxidative stress events in Gram-positive bacteria are diminished.

## Figures and Tables

**Table 1:** Synthesis and characterization of cerium oxide nanoparticles.

Synthesis method	Salt precursor	Particle size			Morphology	Surface area (m^2^/g)	Zeta potential (mV)	Reference
		FDS^A^ (nm)	Electron microscopy (nm)	Others (nm)				
Hydrolysis (Rhodia chemical company)	Ce^4+^(NO_3_^−^)_4_	-	-	7^*∗*^	Ellipsoid	400	Ni	[28]

Hydrolysis (Rhodia chemical company)	Ce^4+^(NO_3_^−^)_4_	-	-	7^*∗*^	Ellipsoid	400	Ni	[29]

Hydrothermal Precipitation	Ce(NO_3_)_3_	-	6 ± 3.5^*¥*^	28.9 ± 18.4^*∗∗*^	Square and oval	Ni	-	[11]
		-	15 ± 4.3^*¥*^	38.1 ± 14.1^*∗∗*^	Circular and oval	Ni	−40–+40	
		-	22.3 ± 5.7^*¥*^	65.7 ± 15.2^*∗∗*^	Oval, rectangular, and triangular	Ni	-	
		-	45 ± 5^*¥*^	126.8 ± 24.1^*∗∗*^	Irregular	Ni	-	

Hydrothermal microwave	Ce(NO_3_)_3_	-	-	7.0^*∗∗*^	-	Ni	20	[7]

Chemical	CeCl_3_	37.6	15–50^*¥*^	-	Ni	Ni	Ni	[15]

Ni (Sigma Aldrich Company)	Ni	-	<25^*¥*^	-	Ni	Ni	Ni	[35]

Precipitation	Ce(NO_3_)_3_	-	-	-	Ni	Ni	Ni	[16]

Precipitation (Flower Extract *Acalypha indica*)	CeCl_3_	36.2	8–54^*¥*^	-	Elliptical and spherical	Ni	Ni	[13]

Sonochemical (Ultrasonication)	Ce(NO_3_)_3_	25	20^*¥*^	-	Cubic	Ni	Ni	[18]

Precipitation *(Aspergillus niger)*	CeCl_3_	14.95	10^*¥*^	-	Cubic and spherical	Ni	Ni	[20]

Precipitation (*Gloriosa superba* L. plant leaf extract)	CeCl_3_	24	5^*¥*^	-	Spherical	Ni	Ni	[21]

Hydrothermal	Ce(NO_3_)_3_	-	25–50^*π*^	-	Spherical	Ni	Ni	[34]

Combustion (Leaf extract *Leucas aspera*)	Ce(NO_3_)_3_	4.3–4.6	-	-	Cubic	Ni	Ni	[22]

Combustion	(NH_4_)_2_Ce(NO_3_)_6_	35	42^*¥*^	-	Spherical	163.5	Ni	[30]

Precipitation (Pectin fruit peel, *Citrus maxima*)	Ce(NO_3_)_3_	23.71	5–40^*π*^	-	Spherical	Ni	−28.0	[23]

Combustion (Watermelon juice Extract)	Ce(NO_3_)_3_	36	-	-	Irregular	Ni	Ni	[17]

Hydrothermal microwave (Peel extract, *Moringa oleifera*)	(NH_4_)_2_Ce(NO_3_)	40–45	45^*¥*^	-	Spherical	Ni	Ni	[14]

^A^Debye-Scherrer formula; ^*∗*^X-ray scattering at a low angle; ^*∗∗*^dynamic scattering of light; Ni: not identified; ^*¥*^transmission electron microscopy; ^*π*^scanning electron microscopy; reference in chronological order; source: original source.

**Table 2:** Recent studies of antimicrobial activity of CeNP against *Staphylococcus aureus*.

*S. aureus* strains	Concentration (mg/mL)	Microbiological technique	Result		Reference^A^
8325-4 CSGR	0.017	CFU count	No significant sensitivity		[7]
	0.17		1.53 ± 0.07 1 g CFU/mL		
	1.72		No significant sensitivity		

ATCC (number ni)	1.37	Time and kill	Inhibition ~40%		[15]
	2.58–3.44		Inhibition > 50%		
	1.37; 2.58 and 5.16	Agar diffusion	Formation of inhibition zone not quantified		

Clinic urinary tract infection	0.05	Agar diffusion	8.00 ± 0.24 mm		[35]
	-	Broth microdilution	Not detected		

ni	Ni (5 mL colloidal solution)	Agar diffusion	17 mm		[13]

ni	10^*∗*^	Agar diffusion	0.0 mm		[21]
	50^*∗*^		~3.33 mm		
	100^*∗*^		5.33 mm		

MSSA ATCC 29213	-	Macrodilution broth	50 ± 20 *µ*g/mL (planktonic culture)	180 ± 80 *µ*g/mL (biofilm)	[34]
MRSA ATCC 43300	-		70 ± 0.0 *µ*g/mL (planktonic culture)	180 ± 80 *µ*g/mL (biofilm)	

NCIM-5022	10 (500 *µ*g/50 *µ*L)	Agar diffusion	1.67 ± 0.33 mm		[22]
	10 (1000 *µ*g/100 *µ*L)		3.33 ± 0.67 mm		

NCIM-5022	10	Agar diffusion	0.0 mm		[30]
	0.2 and 0.4	Diluted in broth	No inhibition		

NCIM-5022	10 (500 *μ*g/50 *μ*L)	Agar diffusion	0.53 ± 0.12 mm		[17]
	10 (1000 *μ*g/100 *μ*L)		1.47 ± 0.03 mm		

Clinical strain	ni (25 *µ*L of solution)	Agar diffusion	5 mm		[14]

CSGR: clinical strain gentamycin-resistant; ^*∗*^mg/disc; Ni: not identified in the paper; MRSA: methicillin-resistant *Staphylococcus aureus*; MSSA: methicillin-sensitive *Staphylococcus aureus*; CFU: colony forming unit; ^A^reference in chronological order. Source: original source.

**Table 3:** Recent studies of antimicrobial activity of CeNP against *Escherichia coli*.

Strain of *E. coli*	Concentration (mg/mL)	Microbiological technique	Result	Reference^A^
RR1	1.0	CFU count	Complete inhibition	[28]

RR1	0.240	CFU count	2% survival	[29]
	-	Fluorescence	Toxicity of approximately 10 ppm with 60% survival	

ATCC 700926	5.0	Agar diffusion	Particle A: <1 mm Particle B: ~3.5 mm Particle C: ~1.8 mm Particle D: ~1 mm	[11]
	0.05; 0.1 and 0.15	Fluorescence	Particle A: between 80 and 90% of viability Particle B: between 35 and 46% of viable cells Particle C: between 60 and 70 percent viable cells Particle D: ~80% of viable cells	
	0.1	Counting CFU/mL	~1 × 10^9^ for groups and controls	

UCM B-930	0.017	CFU count	1.92 ± 0.07 1 g CFU/mL	[7]
	0.17		1.11 ± 0.02 1 g CFU/mL	
	1.72		There was no significant sensitivity	

Clinical urinary tract infection	0.05	Agar diffusion	9.00 ± 0.39 mm	[35]
	-	Broth microdilution	MIC = MBC = 20 *µ*g/mL	

ATCC 25922	3.0	Agar diffusion	0.0 mm	[16]
	3.0 mg/mL sonicated and pH 7		9 mm	
	3.0 mg/mL sonicated and pH 7 + Tween 80		15 mm	
	3.0 mg/mL sonicated and pH 7 + polyvinyl pyrrolidone		14 mm	
	3.0 mg/mL sonicated and pH 7 + Triton-X114		13 mm	
	CeNP with surfactant Tween-80, the 0.001%	Diluted in broth	MIC = 0.15 mg/mL	
	Without surfactant		MIC = 3 mg/mL	

Ni	Ni (5 mL of colloidal solution)	Agar diffusion	9 mm	[13]

KACC 10005	-	Diluted in broth	16 *µ*g/mL	[18]

Ni	1.0	Agar diffusion	0.0 mm	[20]
	5.0		3.33 ± 0.33 mm	
	10.0		6.33 ± 0.33 mm	

Ni	10 mg^*∗*^	Agar diffusion	0.0 mm	[21]
	50 mg^*∗*^		~2.60 mm	
	100 mg^*∗*^		4.0 mm	

ATCC 25922	-	Macrodilution	50 ± 10 *µ*g/mL (planktonic culture) 90 ± 40 *µ*g/mL (biofilm)	[34]

NCIM-5051	10 (500 *μ*g/50 *μ*L)	Agar diffusion	2.67 ± 0.33	[22]
	10 (1000 *μ*g/100 *μ*L)		4.67 ± 0.33	

ATCC 8739	0.17	CFU count	~30% of survival	[23]
	0.34		~5% survival	

Clinical strain	Ni (25 *µ*L of solution)	Agar diffusion	7 mm	[14]

^*∗*^mg/disc; Ni: not identified in the paper; CFU/mL: colony-forming unit per milliliter; MIC: minimum inhibitory concentration; MBC: minimum bactericidal concentration; ^A^reference in chronological order. Source: original source.

**Table 4:** Recent studies of CeNP antimicrobial activity against *Pseudomonas aeruginosa*.

Strain of *P. aeruginosa*	Concentration (mg/mL)	Microbiological technique	Result	Reference^A^
Ni	10^*∗*^	Agar diffusion	0.0 mm	[21]
	50^*∗*^		~3 mm	
	100^*∗*^		4.67 mm	

ATCC 27853	-	Macrodilution	20 ± 5 *µ*g/mL (planktonic culture) 70 ± 0.0 *µ*g/mL (biofilm)	[34]

NCIM-2242	10	Agar diffusion	3.33 mm	[30]
	15		3.57 mm	
	20		4.50 mm	
	0.2 and 0.4	Diluted in broth	Inhibition of growth was observed; MIC was not identified	

^*∗*^mg/paper disk; ni: not identified in the paper; MIC:minimum inhibitory concentration; ^A^reference in chronological order. Source: original source.

**Table 5:** Recent studies of CeNP antimicrobial activity against *Bacillus subtilis*.

Strain of *B. subtilis*	Concentration (mg/mL)	Microbiological technique	Result	Reference^A^
ATCC 6633	5.0	Agar diffusion	Particle A: ~3.2 mm Particle B: <1 mm Particle C: ~2 mm Particle D: ~3 mm	[11]
	0.05; 0.1; 0.15	Fluorescence	Particle A: between 40 and 65% of viable cells Particle B: between 80 and 90% of viable cells Particle C: between 60 and 80% viable cells Particle D: between 45 and 65% of viable cells	
	0.1	Counting CFU/mL	Between 10^8^ and 10^9^ for experimental groups and 10^9^ for control	

KACC 14394	-	Broth microdilution	4 *µ*g/mL	[18]

Ni	1	Agar diffusion	0.0 mm	[20]
	5		4.67 ± 0.33 mm	
	10		10.33 ± 0.33 mm	

ATCC 6633	0.17	CFU count	~40% of survival	[23]
	0.34		~12% of survival	

Ni: not identified in the paper; CFU: colony forming unit; ^A^reference in chronological order. Source: original source.

**Table 6:** Recent studies of CeNP antimicrobial activity against *Proteus*.

Microorganism	Strain of *Proteus*	Concentration (mg/mL)	Microbiological technique	Result	Reference^A^
*Proteus morganii*	Clinical urinary tract infection	0.05	Agar diffusion	11.0 ± 0.51 mm	[35]
		-	Microdilution	MIC = MBC = 20 *µ*g/mL	

*Proteus vulgaris*	Ni	1.0	Agar diffusion	0.0 mm	[20]
		5.0		3.67 ± 0.33 mm	
		10.0		8.33 ± 0.33 mm	

*Proteus vulgaris*	Ni	10^*∗*^	Agar diffusion	0.0 mm	[21]
		50^*∗*^		~3 mm	
		100^*∗*^		4.67 mm	

*Proteus mirabilis*	ATCC 12459	-	Macrodilution	30 ± 10 *µ*g/mL (planktonic culture) 360 ± 160 *µ*g/mL (biofilm)	[34]

^*∗*^mg/disc; Ni: not identified in the paper; MIC: minimum inhibitory concentration; MBC: minimum bactericidal concentration; ^A^reference in chronological order. Source: original source.

**Table 7:** Recent studies of antimicrobial activity of CeNP against *Streptococcus pneumoniae*.

Strain of *S. pneumoniae*	Concentration (mg/mL)	Microbiological technique	Result	Reference^A^
ni	1.0	Agar diffusion	0.0 mm	[20]
	5.0		3.33 ±0.33 mm	
	10.0		10.67 ± 0.33 mm	

ni	10^*∗*^	Agar diffusion	0.0 mm	[21]
	50^*∗*^		~3.60 mm	
	100^*∗*^		~4.33 mm	

ATCC 25923	-	Macrodilution	110 ± 40 *µ*g/mL (planktonic culture) 180 ± 80 *µ*g/mL (biofilm)	[34]

^*∗*^mg/paper disk; ni: not identified in the paper; ^A^reference in chronological order. Source: original source.

## Abbreviations

CeNPs: Cerium oxide nanoparticles
MRSA: Methicillin-resistant* Staphylococcus aureus*
SciELO: Scientific Electronic Library Online
Lilacs: Latin American and Caribbean Literature on Health Sciences
BET: Brunauer-Emmett-Teller
DSF: Debye-Scherrer formula
Ni: Not identified
TEM: Transmission electron microscopy
SEM: Scanning microscopy electron
CFU/mL: Colony forming unit per milliliter
MIC: Minimum inhibitory concentration
MBC: Minimum bactericidal concentration
*E. col*: * Escherichia coli*
*P. aeruginosa*: * Pseudomonas aeruginosa*
*B. subtilis*: * Bacillus subtilis*
*C. albicans*: * Candida albicans*
*E. faecalis*: * Enterococcus faecalis*.
